# Clinical Outcomes for Closure of Iatrogenic Atrial Septal Defects Following Transseptal SAPIEN Mitral Valve-in-Valve Procedures

**DOI:** 10.1016/j.jscai.2025.102636

**Published:** 2025-06-17

**Authors:** Andrew Morse, Samir Kapadia, Mackram Eleid, Susheel K. Kodali, James M. McCabe, Amar Krishnaswamy, Richard Smalling, Mark Reisman, Michael J. Mack, William W. O’Neill, Vinayak N. Bapat, Martin B. Leon, Charanjit S. Rihal, Raj R. Makkar, Mayra E. Guerrero, Brian K. Whisenant, Evelio Rodriguez

**Affiliations:** a Structural Heart & Heart Valve Clinic, Ascension Saint Thomas Heart West, Nashville, Tennessee; b Miller Family Heart, Vascular & Thoracic Institute, Tomsich Family Department of Cardiovascular Medicine, Cleveland Clinic, Cleveland, Ohio; c Department of Cardiovascular Medicine, Mayo Clinic, Rochester, Minnesota; d Columbia Structural Heart and Valve Center, Seymour, Paul and Gloria Milstein Division of Cardiology, Department of Medicine, NewYork-Presbyterian/Columbia University Irving Medical Center, New York, New York; e Division of Cardiology, Department of Medicine, University of Washington Medical Center, Seattle, Washington; f Division of Cardiology, Department of Medicine, McGovern Medical School at UTHealth, Houston, Texas; g Structural Heart Disease Program, Division of Cardiology, Department of Medicine, Weill Cornell Medical Center, New York, New York; h Cardiovascular Surgery, Baylor Scott & White Health, Dallas, Texas; i Heart and Vascular Institute, Center for Structural Heart Disease, Henry Ford Hospital, Detroit, Michigan; j Department of Cardiothoracic Surgery, Allina Health Minneapolis Heart Institute at Abbott Northwestern Hospital, Minneapolis, Minnesota; k Clinical Trials Center, Cardiovascular Research Foundation, New York, New York; l Department of Cardiology, Smidt Heart Institute, Cedars-Sinai Medical Center, Los Angeles, California; m Division of Cardiology, Intermountain Heart Institute, Salt Lake City, Utah; n Cardiovascular Surgery, Ascension Saint Thomas Heart West, Nashville, Tennessee

**Keywords:** dysfunction, iatrogenic atrial septal defect, mitral bioprosthesis, outcomes, transseptal mitral valve-in-valve implantation

## Abstract

**Background:**

Iatrogenic atrial septal defects (iASD) are created during transseptal (TS) mitral valve-in-valve (MViV) implantation to facilitate access. Although most iASD remain untreated, the outcomes of closing iASD during TS MViV are unclear. This study evaluates outcomes of concomitant iASD closure during TS MViV.

**Methods:**

Patients undergoing TS MViV with SAPIEN 3/Ultra/Resilia valves from June 2015 to September 2023 were identified using the Society of Thoracic Surgeons/American College of Cardiology Transcatheter Valve Therapy Registry. To reduce patient selection bias in the primary analysis, MViV patients without iASD closure were chosen from sites that did not perform iASD closures. Propensity score matching accounted for baseline characteristics, and analyses evaluated procedural success, complications, and 1-year clinical outcomes.

**Results:**

Among 5363 TS MViV patients, 472 (8.8%) underwent iASD closure at 173 of 494 sites (35.0%). Propensity matching yielded 468 patient pairs (34% male, 66% female). No significant differences were observed in procedural success, complications, stroke (3.3% vs 5.2%; *P* = .26), or mortality (18.8% vs 17.3%; *P* = .54). Rates of New York Heart Association class III/IV and heart failure rehospitalization were also similar. However, in patients with severe pulmonary hypertension (mean pulmonary artery pressure, 47.4 ± 8.6 mm Hg), iASD closure was associated with higher 30-day mortality (9.7% vs 3.9%; *P* = .03) and 1-year cardiac readmission rates (14.1% vs 4.1%; *P* = .008).

**Conclusions:**

Iatrogenic atrial septal defect closure during the index hospitalization for TS MViV patients is a well-tolerated procedure when performed in carefully selected individuals. However, no significant clinical benefits were observed in the iASD closure group. Additionally, patients with significant pulmonary hypertension did not demonstrate any clinical advantage from iASD closure, and the procedure may even pose potential harm in this subgroup.

## Introduction

Reoperation of failed bioprosthetic mitral valves poses significant morbidity and mortality, leading to the adoption of transcatheter mitral valve-in-valve (MViV) therapy as an alternative for high-risk patients.^1^ Approved by the FDA in 2017, MViV procedures have gained widespread adoption, with the transseptal (TS) approach becoming the preferred method due to its less invasive nature.^2^ This approach involves creating an iatrogenic atrial septal defect (iASD) via balloon septostomy, which may require closure in certain cases. The Society of Thoracic Surgeons/American College of Cardiology Transcatheter Valve Therapy (STS/ACC TVT) Registry monitors MViV outcomes, with prior studies demonstrating high success rates and low mortality. Currently, the SAPIEN 3 and SAPIEN Ultra valves are the primary devices approved for MViV procedures.^3^^,^^4^ Several prior publications, including Whisenant et al^4^ in 2020 have demonstrated high technical success and low 30-day/1-year mortality of this therapy.^5^^,^^6^ Although non–balloon septostomy iASD often close spontaneously, the clinical impact of balloon septostomy iASD, especially those closed during the procedure, remains unclear. Using the TVT Registry linked with CMS claims data, this study assesses the outcomes of iASD closure during MViV, addressing an unexplored aspect of this therapy.

## Methods

This retrospective analysis included all patients who underwent TS MViV using balloon-expandable valves (BEV; SAPIEN 3, SAPIEN 3 Ultra, or SAPIEN 3 Ultra Resilia) with or without an iASD closure in the STS/ACC TVT Registry and CMS claims data from June 2015 to September 2023. Standardized definitions of adverse events and outcomes were based on the TVT Registry Data Coder Dictionary.^7^ Baseline characteristics, peri-procedural, in-hospital, 30-day, and 1-year clinical outcomes were derived from the TVT Registry and CMS claims data. CMS claims data were available for all-cause mortality and stroke outcomes. The TVT Registry was linked to CMS claims data using probabilistic matching with patient birth date, sex, and TAVR procedure date. Patients eligible for linkage were 65 years or older with Medicare coverage and enrolled in the Medicare Parts A and B fee-for-service program.

In the primary analysis, to minimize the impact of patient selection bias, MViV patients who did not undergo iASD closure were selected from sites that did not perform any iASD closures for MViV procedures during the study. In a sensitivity analysis, MViV patients who did not undergo iASD closure were selected from sites that did perform iASD closures for MViV procedures during the study period.

### Objectives and end points

The primary objective of the study was to assess the clinical outcomes of patients undergoing iASD closure at the time of the MViV procedure compared to those who did not. The primary efficacy end point was all-cause mortality at 1 year. Procedural technical success was assessed by Mitral Valve Academic Research Consortium criteria at the exit from the catheterization laboratory or hybrid suite (patient alive with successful access, delivery, retrieval of the device delivery system, successful deployment, correct position of the first intended device, and freedom from emergency surgery or reintervention associated with the device or access procedure). The secondary 30-day and 1-year outcome analysis included cardiovascular death, stroke, mitral valve reintervention, readmission for heart failure, New York Heart Association (NYHA) class, quality of life defined by the 12-item Kansas City Cardiomyopathy Questionnaire Overall Summary (KCCQ-OS) score, echocardiographic outcomes, and adverse events. Clinical outcomes of a subanalysis of pulmonary hypertension patients with mean pulmonary arterial pressure (mPAP) of ≥35 mm Hg are also reported.

Edwards Lifesciences performed the analyses using data from the TVT Registry. The authors had control of data analyses and the manuscript contents.

### Statistical analysis

Patients who underwent MViV with iASD closure were propensity score–matched with patients who underwent MViV without iASD closure using 41 covariates to minimize potential selection bias related to differences in baseline characteristics between TS MViV patients. The covariates included age, sex, body mass index, STS score, access site, prior percutaneous coronary intervention, prior coronary artery bypass graft surgery, prior stroke, carotid stenosis, peripheral arterial disease, hypertension, diabetes, chronic lung disease, immunocompromised, porcelain aorta, atrial fibrillation, creatinine, hemoglobin level, estimated glomerular filtration ratio, left ventricular ejection fraction, moderate or greater aortic regurgitation, moderate or greater mitral regurgitation (MR), moderate or greater tricuspid regurgitation (TR), NYHA functional class III/IV, KCCQ-OS score, currently on dialysis, pacemaker, previous implantable cardioverter-defibrillator, cardiogenic shock within 24 hours, prior transient ischemic attack, endocarditis, hostile chest, prior myocardial infarction, mitral valve area, mitral annulus calcification, B-type natriuretic peptide (BNP), N-terminal-pro hormone BNP, pulmonary vascular resistance, pulmonary artery pressure, pulmonary artery pressure (systolic), and mean right atrial pressure/central venous pressure. Missing baseline values were imputed using the Markov Chain Monte Carlo method before modeling. The balance between cohorts was determined by calculating standardized differences in which a difference of less than 0.10 was considered as achieving a good balance.

Continuous variables were presented as mean ± SD or median (IQR) and were compared between groups using the 2-sample *t* test or Wilcoxon rank sum test. Categorical variables were given as frequencies and percentages. These were compared using the χ^2^ or Fisher exact test. The 30-day and 1-year adverse event rates were based on Kaplan-Meier estimates and all comparisons were made using the log-rank test. All statistical analyses were performed using SAS version 9.4 (SAS Institute), and statistical significance was set at a 2-sided *P* < .05 without multiplicity adjustment.

## Results

### Registry population and baseline characteristics

**Figure 1:**
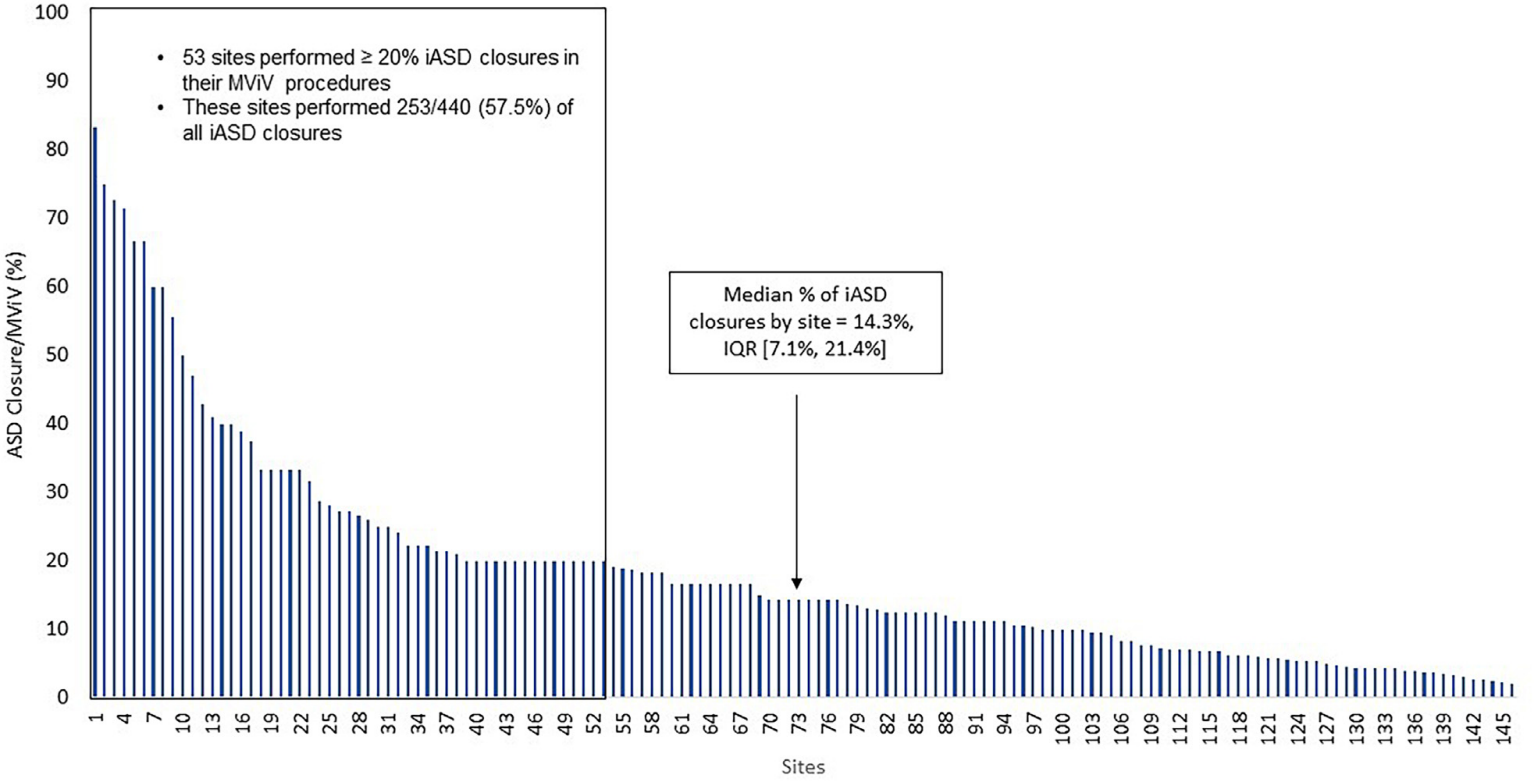
**Percentage of iatrogenic atrial septal defect (iASD) closure over mitral valve-in-valve (MViV) procedure among sites.** Sites that performed at least 1 iASD closure and 5 MViV procedures over the study period were included..

**Figure 2:**
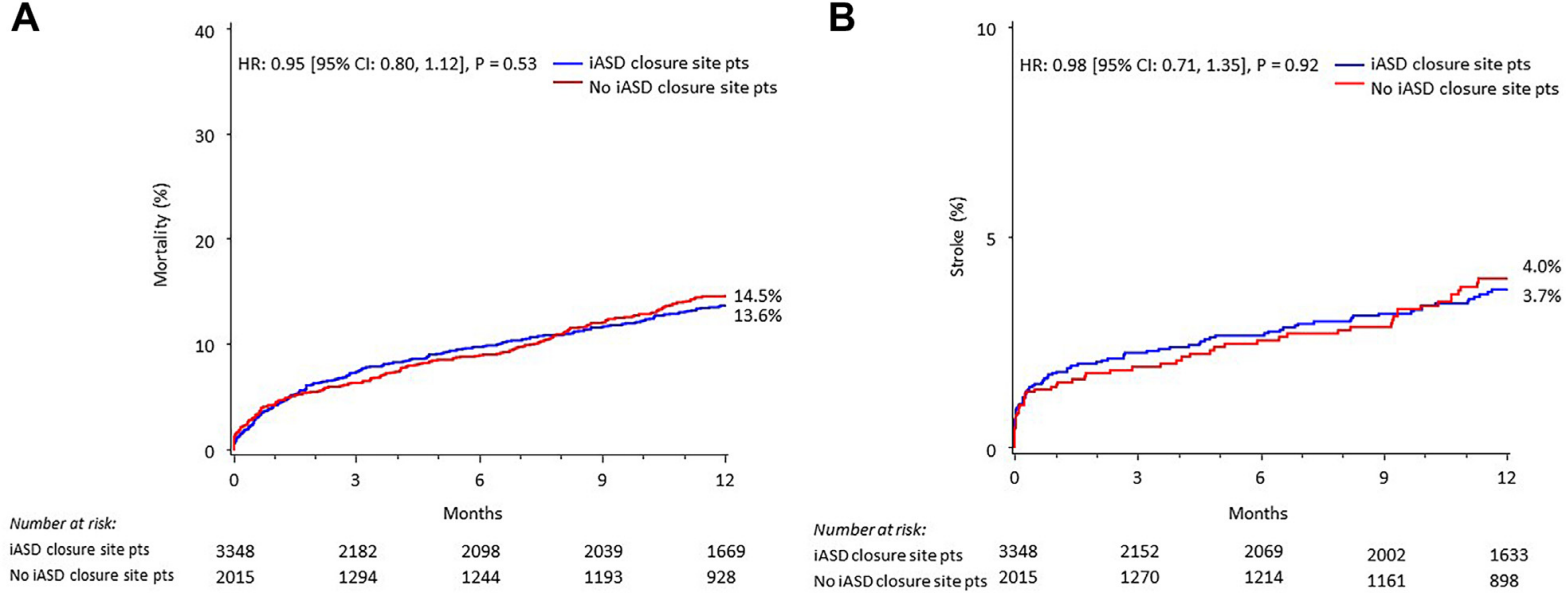
**One-year Kaplan-Meier all-cause mortality and stroke curves in iatrogenic atrial septal defect (iASD) closure site vs no iASD closure site mitral valve-in-valve (MViV) patients (pts).** Kaplan-Meier estimates of all-cause mortality (**A**) and stroke (**B**) to 1 year in iASD closure site pts vs no iASD closure site pts who underwent MViV. HR, hazard ratio.

A total of 5837 patients underwent SAPIEN 3/Ultra/Resilia transcatheter MViV procedures between June 2015 and September 2023 at 506 sites in the US (Central Illustration). After study exclusions, there were 5363 patients who underwent MViV with the TS approach, of which 3348 procedures were from sites that performed iASD closures and 2015 procedures were from sites that did not perform iASD closures. iASD closures were performed at a frequency of 8.8% (472/5363) of all TS MViV procedures. However, procedural frequency varied greatly across sites, with frequencies greater than 60% in 6 sites, including 1 site that performed closure in greater than 80% of all their TS MViV procedures (Figure 1). The median percentage of iASD closures was 14.3% (7.1%, 21.4%). Fifty-three sites performed ≥20% iASD closures in their MViV procedures, representing 57.5% (253/440) of all iASD closures. There were no differences in 1-year mortality and stroke rates in patients from sites that performed iASD closures vs patients from sites that did not perform any iASD closures (Figure 2).

**Central Illustration:**
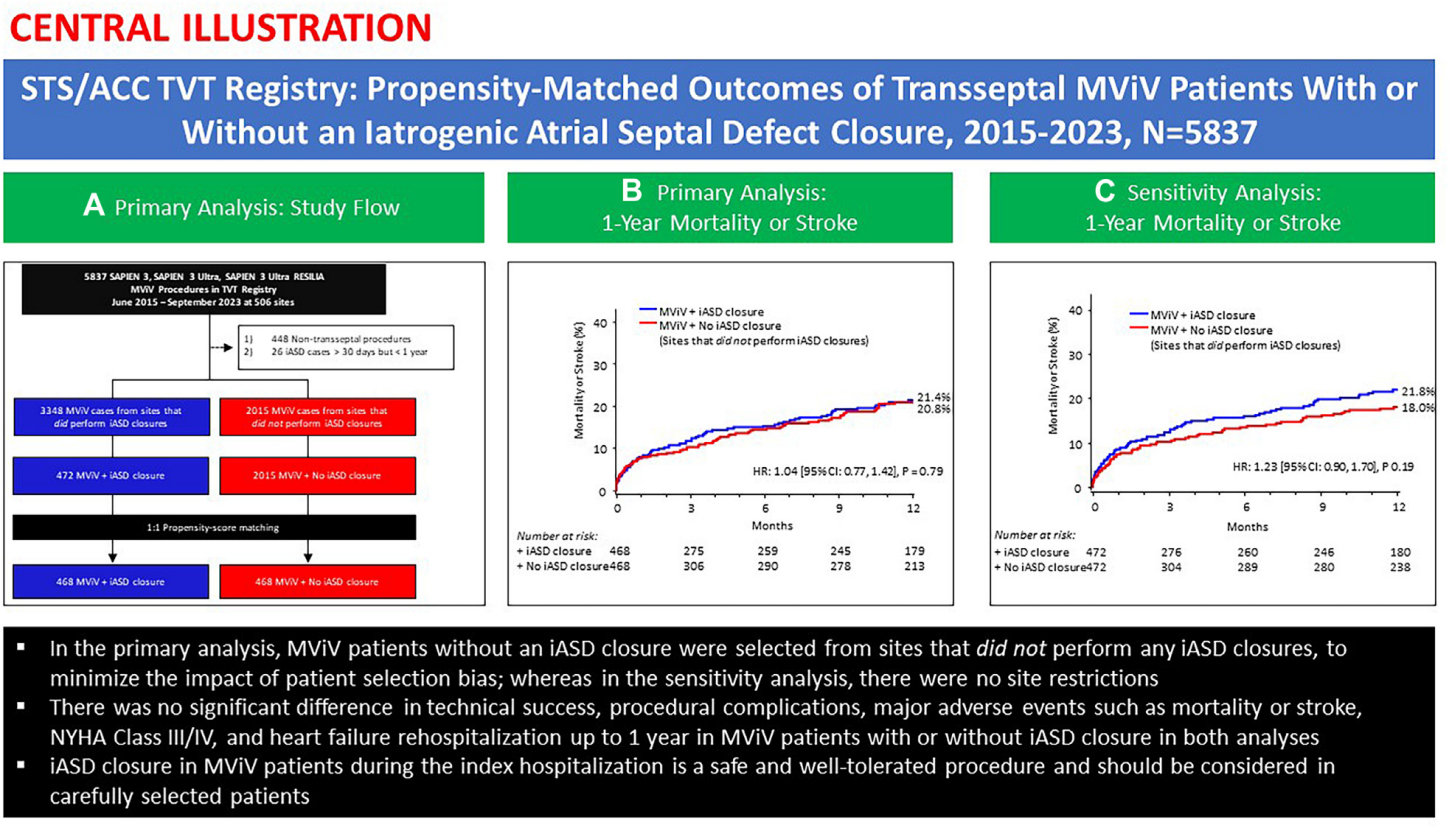
One-year outcomes of transseptal mitral valve-in-valve (MViV) patients with or without an iatrogenic atrial septal defect (iASD) closure using balloon-expandable valves are shown. (A) Primary analysis: study flow; (B) Primary analysis: 1-year all-cause mortality or stroke curves in patients who underwent MViV with or without iASD closure (in sites that did not perform iASD closures); (C) 1-year all-cause mortality or stroke curves in patients who underwent MViV with or without iASD closure (in sites that did perform iASD closures). HR, hazard ratio; NYHA, New York Heart Association; STS/ACC TVT, Society of Thoracic Surgeons/American College of Cardiology Transcatheter Valve Therapy.

The TS MViV patients treated at iASD-performing sites were utilized to compare outcomes between those who underwent iASD closure (n = 472) and those who did not at sites that did not perform closures (n = 2015). Before propensity score matching, patients who underwent iASD closure comprised a cohort with overall greater clinical acuity, characterized by elevated surgical risk (STS score), increased incidence of atrial fibrillation/flutter, and a higher likelihood for chronic lung disease, and a trend toward higher NYHA class III/IV. Moreover, this group presented with a clinical profile marked by higher rates of cardiogenic shock, elevated levels of BNP, and renal dysfunction. Baseline echocardiographic findings were consistent with these clinical presentations, demonstrating a higher prevalence of moderate to severe regurgitation (mitral and tricuspid), and elevated right heart pressures (right atrial and pulmonary artery pressures; Table 1).

**Table 1:** Unmatched and matched baseline clinical and echocardiographic characteristics.

	All MViV patients (N = 2487)	Unmatched MViV + ASD closure (n = 472)	Unmatched MViV + no ASD Closure (n = 2015)	*P* value	Matched MViV + ASD closure (n = 468)	Matched MViV + no ASD closure (n = 468)	*P* value
Clinical characteristics							
Age, y	73.4 ± 11.0 (2487)	72.9 ± 11.6 (472)	73.5 ± 10.9 (2015)	.30	73.0 ± 11.6 (468)	72.5 ± 11.2 (468)	.55
STS risk score, %	9.2 ± 7.8 (2328)	10.5 ± 9.0 (438)	8.9 ± 7.5 (1890)	.0007	10.5 ± 9.0 (436)	10.3 ± 8.9 (424)	.81
Male	40.9 (1018/2487)	34.5 (163/472)	42.4 (855/2015)	.002	34.4 (161/468)	34.2 (160/468)	.95
Female	59.1 (1469/2487)	65.5 (309/472)	57.6 (1160/2015)	.002	65.6 (307/468)	65.8 (308/468)	.95
BMI, kg/m^2^	27.4 ± 7.5 (2480)	27.3 ± 6.7 (472)	27.5 ± 7.6 (2008)	.67	27.3 ± 6.7 (468)	27.6 ± 8.5 (465)	.55
Hypertension	85.0 (2114/2486)	87.5 (413/472)	84.5 (1701/2014)	.10	87.4 (409/468)	87.8 (411/468)	.84
Diabetes	27.7 (688/2484)	28.8 (136/472)	27.4 (552/2012)	.55	28.6 (134/468)	28.9 (135/467)	.93
Currently on dialysis	5.4 (135/2482)	6.8 (32/472)	5.1 (103/2010)	.15	6.6 (31/468)	5.8 (27/464)	.61
Peripheral arterial disease	13.5 (334/2484)	15.1 (71/471)	13.1 (263/2013)	.25	15.0 (70/467)	16.5 (77/468)	.54
Home oxygen	14.5 (359/2482)	15.7 (74/471)	14.2 (285/2011)	.39	15.9 (74/467)	14.1 (66/467)	.46
Cardiogenic shock within 24 h	4.1 (101/2486)	8.9 (42/472)	2.9 (59/2014)	<.0001	8.1 (38/468)	8.3 (39/468)	.91
Carotid stenosis	9.9 (209/2122)	9.6 (38/396)	9.9 (171/1726)	.85	9.7 (38/392)	10.2 (41/401)	.80
Atrial fibrillation/flutter	72.7 (1809/2487)	78.4 (370/472)	71.4 (1439/2015)	.002	78.4 (367/468)	76.1 (356/468)	.39
Prior stroke	17.7 (440/2487)	16.7 (79/472)	17.9 (361/2015)	.55	16.7 (78/468)	16.2 (76/468)	.86
Chronic lung disease	38.9 (964/2479)	43.1 (203/471)	37.9 (761/2008)	.04	42.6 (199/467)	43.0 (200/465)	.90
Prior PCI	16.9 (421/2486)	18.4 (87/472)	16.6 (334/2014)	.34	18.2 (85/468)	16.0 (75/468)	.39
Prior CABG	30.1 (749/2485)	30.2 (142/471)	30.1 (607/2014)	>.99	30.4 (142/467)	31.8 (149/468)	.64
Porcelain aorta	0.3 (8/2487)	0 (0/472)	0.4 (8/2015)	.37	0 (0/468)	0 (0/468)	—
BNP	838.1 ± 1110.6 (959)	1019.8 ± 1171.6 (208)	787.7 ± 1088.5 (751)	.008	1019.8 ± 1171.6 (208)	961.0 ± 1319.8 (174)	.64
GFR, mL/min/1.73 m^2^	55.5 ± 24.9 (2483)	53.0 ± 29.3 (471)	56.1 ± 23.7 (2012)	.04	53.2 ± 29.4 (467)	52.6 ± 24.0 (466)	.73
Hemoglobin	11.5 ± 2.0 (2483)	11.2 ± 2.0 (471)	11.6 ± 2.0 (2012)	.001	11.2 ± 2.0 (467)	11.2 ± 2.0 (467)	.79
NYHA III/IV	82.6 (2024/2451)	85.7 (402/469)	81.8 (1622/1982)	.05	85.6 (398/465)	84.5 (388/459)	.65
KCCQ-OS score	35.8 ± 23.4 (2112)	32.2 ± 23.0 (380)	36.7 ± 23.4 (1732)	.0007	32.3 ± 22.9 (378)	33.6 ± 23.2 (386)	.43
Echocardiography characteristics							
Left ventricular ejection fraction, %	54.6 ± 11.8 (2462)	54.6 ± 12.2 (470)	54.6 ± 11.7 (1992)	.97	54.6 ± 12.2 (466)	54.7 ± 12.2 (461)	.90
Mitral stenosis	77.0 (1868/2425)	78.1 (363/465)	76.8 (1505/1960)	.56	78.1 (360/461)	72.0 (322/447)	.03
Mitral mean valve gradient, mm Hg	13.0 ± 5.8 (2368)	13.6 ± 5.8 (457)	12.9 ± 5.8 (1911)	.02	13.5 ± 5.8 (453)	13.3 ± 6.0 (443)	.54
Mitral valve area, cm^2^	1.4 ± 1.0 (1882)	1.3 ± 0.9 (358)	1.4 ± 1.0 (1524)	0.03	1.3 ± 0.9 (355)	1.5 ± 1.0 (348)	.07
≥ Moderate aortic regurgitation	9.5 (233/2453)	8.7 (41/469)	9.7 (192/1984)	.53	8.6 (40/465)	8.0 (37/460)	.76
≥ Moderate mitral regurgitation	52.6 (1299/2469)	57.0 (268/470)	51.6 (1031/1999)	.03	57.1 (266/466)	58.6 (272/464)	.63
≥ Moderate tricuspid regurgitation	58.3 (1441/2474)	68.3 (319/467)	55.9 (1122/2007)	<.0001	68.0 (315/463)	68.0 (317/466)	>.99
Pulmonary capillary wedge pressure, mm Hg	26.4 ± 8.5 (1268)	26.7 ± 9.3 (259)	26.3 ± 8.3 (1009)	.52	26.5 ± 9.2 (255)	28.0 ± 8.5 (234)	.07
Pulmonary artery pressure (mean), mm Hg	41.0 ± 12.3 (1297)	42.5 ± 11.6 (261)	40.6 ± 12.4 (1036)	.03	42.3 ± 11.5 (257)	42.5 ± 13.3 (241)	.88
Pulmonary artery pressure (systolic), mm Hg	63.7 ± 18.6 (1395)	65.8 ± 18.2 (288)	63.1 ± 18.6 (1107)	.03	65.6 ± 18.1 (284)	65.3 ± 18.5 (262)	.88
Right atrial pressure/central venous pressure, mm Hg	12.4 ± 6.8 (1354)	14.5 ± 7.3 (280)	11.8 ± 6.5 (1074)	<.0001	14.3 ± 7.2 (276)	13.8 ± 7.2 (252)	.40
Pulmonary vascular resistance	319.7 ± 305.8 (1096)	354.5 ± 297.3 (229)	310.5 ± 307.5 (867)	.05	346.8 ± 276.6 (225)	371.6 ± 500.5 (199)	.54

Primary analysis: MViV + ASD closure vs MViV + no ASD closure (in sites that did not perform ASD closures). Values are mean ± SD (n) or % (n/N).

ASD, atrial septal defect; BMI, body mass index; BNP, B-type natriuretic peptide; CABG, coronary artery bypass grafting; GFR, glomerular filtration rate; KCCQ-OS, Kansas City Cardiomyopathy Questionnaire Overall Summary; MViV, mitral valve-in-valve; NYHA, New York Heart Association; PCI, percutaneous coronary intervention; STS, Society of Thoracic Surgeons.

After performing propensity score matching, 472 matched pairs were analyzed. No significant differences were observed in baseline demographic or echocardiographic parameters between the matched groups (Table 1). Similarly, among sites that performed iASD closures, no differences in baseline demographic or echocardiographic outcomes were identified between propensity score–matched patients who underwent iASD closure and those who did not (472 matched pairs; Supplemental Table S1).

An additional subgroup analysis was performed on patients with severe pulmonary hypertension, defined as those with an mPAP of ≥35 mm Hg. These patients underwent invasive right heart catheterization, which revealed hemodynamic parameters indicative of severe disease, including mPAP of 47.4 ± 8.6 mm Hg, mean right atrial pressures of 16.0 ± 6.6 mm Hg, and pulmonary vascular resistance of 414.7 ± 279.6 dynes/s/cm^–5^ in the ASD closure group. Within this subgroup, propensity score matching resulted in 184 matched pairs. No significant differences were observed in baseline clinical or echocardiographic parameters between patients who underwent iASD closure and those who did not at sites performing these procedures (Supplemental Table S2).

### Procedural and in-hospital outcomes

In the iASD closure versus the no iASD closure patients, the Mitral Valve Academic Research Consortium technical success was similar (95.7% vs 97.0%; *P* = .38) between the groups (Table 2). There was no difference in procedural outcomes, including implant success, procedure status, conversion to open heart surgery, device embolization, device thrombosis, or cardiac perforation. Although the procedural times were similar between the groups, the iASD closure cohort had a trend toward increased procedural time (116.5 ± 64.7 vs 108.8 ± 66.8 minutes; *P* = .07) and significantly increased fluoroscopy times (37.4 ± 23.7 vs 32.3 ± 18.0 minutes; *P* = .0005). The overall risk of all-cause in-hospital mortality, stroke, and vascular complications was similar between the groups. There was no difference in the rate of discharge to home. However, there was a slightly longer median length of stay (3.0 vs 2.0; *P* = .01). These findings were comparable in the propensity score–matched analysis for iASD closure patients versus no iASD closure patients from sites that did perform iASD closures (Supplemental Table S3).

**Table 2:** Propensity-matched procedural and in-hospital outcomes.

	MViV + ASD closure (n = 468)	MViV + no ASD closure (n = 468)	*P* value
Procedural outcomes			
MVARC technical success^a^	95.7 (448/468)	97.0 (454/468)	.38
Procedure status			
Elective	62.8 (294/468)	65.0 (304/468)	.50
Urgent	33.6 (157/468)	29.1 (136/468)	.14
Emergency	2.6 (12/468)	4.7 (22/468)	.08
Salvage	1.1 (5/468)	1.3 (6/468)	.76
THV type			
SAPIEN 3 Ultra Resilia	7.1 (33/468)	4.3 (20/468)	.07
SAPIEN 3 Ultra	27.1 (127/468)	27.1 (127/468)	1.00
SAPIEN 3	65.8 (308/468)	68.6 (321/468)	.37
THV size			
20 mm	0 (0/468)	0 (0/467)	—
23 mm	6.8 (32/468)	9.0 (42/467)	.22
26 mm	38.5 (180/468)	45.4 (212/467)	.03
29 mm	54.7 (256/468)	45.6 (213/467)	.005
Device implant success	96.4 (451/468)	97.4 (456/468)	.35
Procedure time, min	116.5 ± 64.7 (467)	108.8 ± 66.8 (468)	.07
Fluoroscopy time, min	37.4 ± 23.7 (416)	32.3 ± 18.0 (420)	.0005
Procedure aborted	0.2 (1/468)	0.6 (3/468)	.62
Converted to open heart surgery	0.6 (3/468)	1.1 (5/468)	.73
Cardiopulmonary bypass	1.1 (5/438)	1.6 (7/444)	.58
Mechanical support	5.6 (26/468)	5.8 (27/467)	.88
Device embolization	0.4 (2/468)	0.0 (0/468)	.50
Device thrombosis	0.2 (1/468)	0.0 (0/468)	1.00
In-hospital outcomes			
All-cause death	4.9 (23/468)	4.7 (22/468)	.88
Cardiovascular death	2.8 (13/468)	2.4 (11/468)	.68
Stroke	1.3 (6/468)	1.7 (8/468)	.59
Mitral valve reintervention	0.6 (3/468)	0.4 (2/468)	1.00
LVOT obstruction	0.9 (4/468)	0.4 (2/468)	.69
New pacemaker without baseline pacemaker	1.6 (5/320)	0.3 (1/313)	.22
Periprocedural myocardial infarction	0.2 (1/468)	0.0 (0/468)	1.00
Device thrombosis	0.2 (1/468)	0.0 (0/468)	1.00
Major vascular complication	1.3 (6/468)	1.7 (8/468)	.59
Cardiac perforation	0.2 (1/468)	1.1 (5/468)	.22
Length of stay, d	3.0 (1.0-7.0)	2.0 (1.0-5.0)	.009
Discharged home	78.6 (368/468)	81.8 (383/468)	.22
Discharge medication			
Antiplatelet	66.8 (292/437)	72.8 (321/441)	.05
Anticoagulant	85.4 (373/437)	83.5 (368/441)	.44

Primary analysis: MViV + ASD closure vs MViV + no ASD Closure (in sites that did not perform ASD closures). Values are mean ± SD (n), % (n/N), or median (IQR).

ASD, atrial septal defect; IQR, interquartile range; LVOT, left ventricular outflow tract; MVARC, Mitral Valve Academic Research Consortium; MViV, mitral valve-in-valve; THV, transcatheter heart valve.

a MVARC technical success was defined as at exit from the hybrid suite, the patient is alive with successful access, delivery, and retrieval of the device delivery system, successful deployment and correct position of the first intended device, and freedom from emergency surgery or reintervention associated with the device or access procedure.

In the pulmonary hypertension subgroup analysis, there was a higher rate of TS complications noted in the iASD group compared to the no iASD closure group (4.3% vs 0.5%; *P* = .04). However, there were no differences between the acute procedure or in-hospital outcomes of these patients (Supplemental Table S4).

### Clinical outcomes at 30 days and 1 year

At 30 days and 1 year, there were no differences in most serious adverse events between those who received iASD closure versus those who did not from sites that did not perform any closures. Most patients in both groups had similar significant clinical improvement from baseline with improvement in NYHA class and KCCQ-OS scores (Table 3). Echocardiography follow-up at 1 year also demonstrated no difference in left ventricular ejection fraction, moderate or greater TR, or valve performance (mean mitral valve gradient and moderate or greater MR).

**Table 3:** Propensity-matched 30-day and 1-year outcomes.

	30-day MViV + ASD closure (n = 468)	30-day MViV + no ASD closure (n = 468)	*P* value	1-year MViV + ASD closure (n=468)	1-Year MViV + no ASD closure (n = 468)	*P* value
Clinical outcomes						
All-cause death	6.6 (30)	5.7 (26)	.58	18.8 (69)	17.3 (66)	.53
Cardiac death	3.1 (14)	2.9 (13)	.85	6.0 (22)	5.2 (20)	.70
Observed: expected ratio	0.66	0.6		NA		
Stroke	1.7 (8)	2.4 (11)	.49	3.3 (12)	5.2 (19)	.26
Mitral valve reintervention	0.9 (4)	0.7 (3)	.70	1.3 (5)	1.0 (4)	.71
Readmission for heart failure	3.9 (17)	2.8 (12)	.34	13.0 (43)	11.2 (37)	.37
New requirement for dialysis	2.3 (10)	1.1 (5)	.19	2.5 (11)	1.1 (5)	.13
Major vascular complication	1.3 (6)	2.2 (10)	.32	1.6 (7)	2.2 (10)	.47
Myocardial infarction	0.5 (2)	0.0 (0)	.16	0.9 (3)	1.2 (3)	.93
New onset of atrial fibrillation	1.5 (7)	0.4 (2)	.10	2.3 (10)	1.0 (4)	.11
New pacemaker without baseline pacemaker	1.6 (5)	0.3 (1)	.11	2.8 (7)	2.0 (4)	.35
Device thrombosis	0.5 (2)	0.0 (0)	.16	0.8 (3)	0.0 (0)	.08
NYHA III/IV	12.8 (33/258)	17.5 (52/297)	.12	13.4 (18/134)	17.3 (29/168)	.36
KCCQ improvement from baseline	35.0 ± 28.8 (248)	36.9 ± 27.7 (268)	.44	42.5 ± 28.5 (115)	38.5 ± 28.6 (143)	.27
Echocardiographic outcomes						
Left ventricular ejection fraction, %	53.9 ± 11.6 (307)	53.8 ± 11.8 (300)	.93	53.3 ± 12.4 (115)	54.5 ± 10.6 (158)	.38
Mean mitral valve gradient, mm Hg	7.7 ± 3.0 (302)	7.7 ± 3.7 (297)	.99	7.5 ± 3.0 (108)	7.4 ± 2.9 (146)	.80
Mitral valve area, cm^2^	1.9 ± 1.2 (174)	2.1 ± 1.1 (172)	.19	1.9 ± 1.2 (56)	1.8 ± 0.9 (74)	.54
≥ Moderate mitral regurgitation	1.0 (3/306)	1.0 (3/301)	1.00	0.0 (0/113)	0.0 (0/150)	N/A
≥ Moderate tricuspid regurgitation	45.9 (141/307)	50.5 (149/295)	.26	36.3 (41/113)	37.0 (57/154)	.90

Primary analysis: MViV + ASD closure vs MViV + no ASD Closure (in sites that did not perform ASD closures). Values are Kaplan-Meier estimate % (no. of events), mean ± SD (n), or % (n/N).

ASD, atrial septal defect; KCCQ, Kansas City Cardiomyopathy Questionnaire; MViV, mitral valve-in-valve; NA, not applicable; NYHA, New York Heart Association.

**Figure 3:**
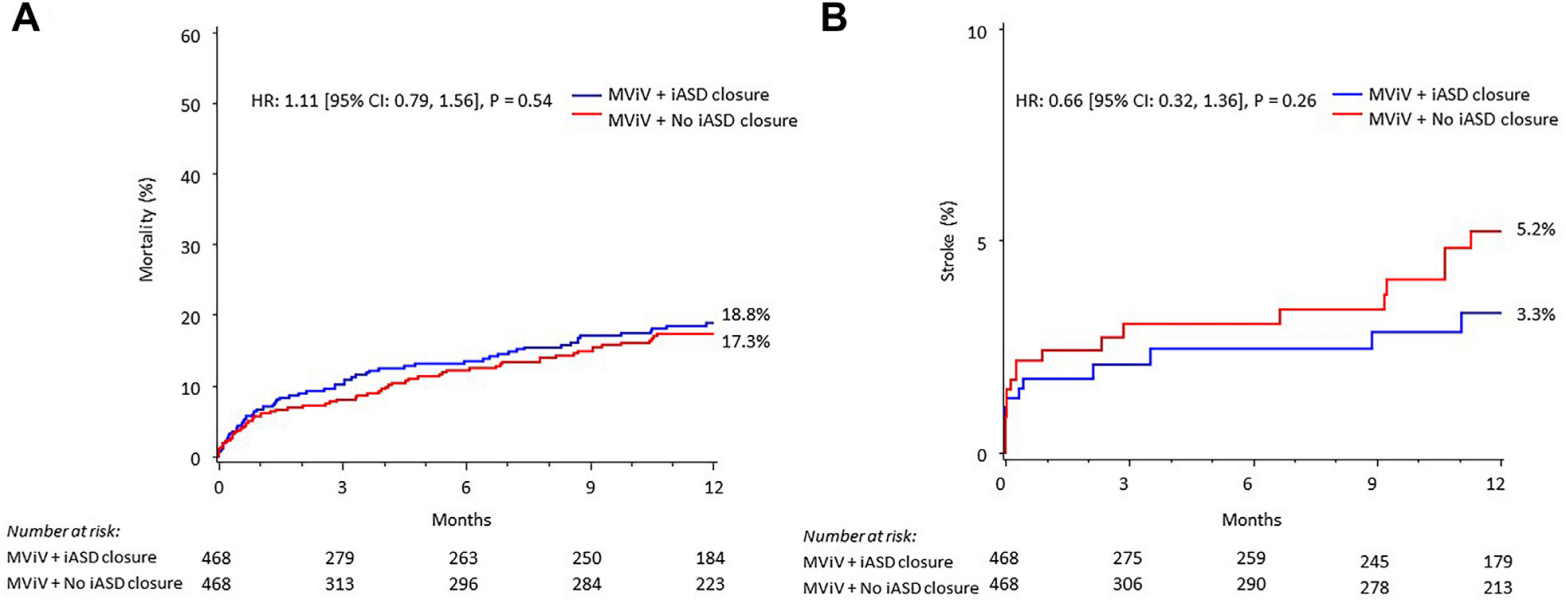
**Primary analysis: 1-year Kaplan-Meier all-cause mortality and stroke curves.** Kaplan-Meier estimates of all-cause mortality (**A**) and stroke (**B**) to 1 year in mitral valve-in-valve (MViV) patients with or without iatrogenic atrial septal defect (iASD) closure (in sites that did not perform iASD closures). CI, confidence interval; HR, hazard ratio.

**Supplemental Figure 1:**
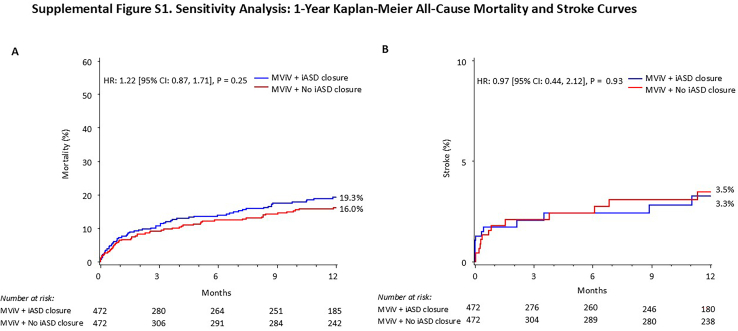
Sensitivity Analysis: 1-Year Kaplan-Meier All-Cause Mortality and Stroke Curves. Kaplan-Meier estimates of all-cause mortality (A) and stroke (B) to 1 year in MViV patients with or without iASD closure (in sites that *did* perform iASD closures). Abbreviations: HR, hazard ratio; iASD, iatrogenic atrial septal defect; MViV, mitral valve-in-valve

There were no significant differences in 1-year Kaplan-Meier estimates of all-cause mortality and stroke for cohorts with or without iASD closure (Figure 3). One-year mortality rate was 18.8% for the iASD closure group and 17.3% for the no iASD closure group (*P* = .54). However, the 1-year stroke rate was 3.3% for the iASD closure group and 5.2% for the no iASD closure group (*P* = .26). Similar results were observed when comparing iASD closure patients to the no iASD closure patients from sites that did perform iASD closures (Supplemental Table S5 and Supplemental Figure S1). Moreover, there was no significant difference in the 1-year composite of mortality or stroke in patients with or without iASD closure in sites that both did and did not perform iASD closures (Central Illustration).

In the propensity-matched pulmonary hypertension subanalysis (defined as mPAP ≥35 mm Hg), a significant difference in 30-day mortality was observed between patients who underwent iASD closure and those who did not (9.7% vs 3.9%; *P* = .03). However, no significant differences were noted in other serious adverse events between the groups at 30 days.

**Supplemental Figure 2:**
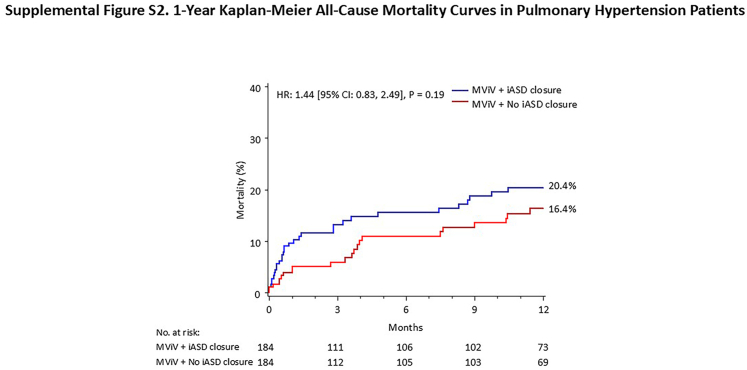
1-Year Kaplan-Meier All-Cause Mortality Curves in Pulmonary Hypertension Patients Kaplan-Meier estimates of all-cause mortality to 1 year in MViV patients with mean pulmonary artery pressure ≥35 mm Hg with or without iASD closure (in sites that did not perform iASD closures). Abbreviations: HR, hazard ratio; iASD, iatrogenic atrial septal defect; MViV, mitral valve-in-valve.

At 1 year, there were no significant differences in the Kaplan-Meier estimates of all-cause mortality. Notably, cardiac readmissions were significantly higher in the iASD closure group compared to the no iASD closure group (14.1% vs 4.1%; *P* = .008). Beyond these findings, no additional differences in serious adverse events were observed at either 30 days or 1 year (Supplemental Tables S6 and S7, Supplemental Figure S2).

## Discussion

We used propensity score matching to eliminate statistically significant demographic and clinical variables to reduce bias in the comparison of patients with iASD closure to those without closure at the time of the MViV procedure. There were no differences in survival, stroke, heart failure readmissions, severity of TR, or valve performance on follow-up in our analysis. Our analysis demonstrates that the procedural, in-hospital, 30-day, and 1-year clinical and echocardiographic outcomes are similar between both groups. Furthermore, iASD closure is a safe and well-tolerated procedure when done during the index hospitalization for an MViV procedure. More clinical research has previously focused on iASD closure in the context of transcatheter edge-to-edge repair (TEER) to date. However, iASD closure in the context of TS MViV has been increasing.^3^ Furthermore, it may be more relevant given the larger size of the iASD that is typically created with MViV procedures as compared to TEER. To our knowledge, our study has the largest cohort considering patients with iASD closure post-TS MViV that has been reported. In our analysis, we restricted the no iASD closure control group to sites that, as a rule, did not perform any iASD closures at all in an attempt to eliminate selection-based bias in cardiovascular health. This approach reduces the variation of entering patients selected for closure, effectively increasing the objectivity of evaluating the impact of iASD closure. There was a lower rate of stroke observed in patients who underwent iASD closure, but this was not statistically significant compared to patients who did not have closure (Figure 3B; 3.3% vs 5.2%; *P* = .26). Similarly, the evaluation of patients who underwent iASD closure by this approach yielded an identical lack of any statistically significant difference by any metric, including procedural success, major complications, length of stay, mortality (1-year, 18.8% vs 17.3% with no iASD closure; *P* = .53), cardiac mortality, heart failure rehospitalization (1-year, 13.0% vs 11.2% with no iASD closure; *P* = .37), NYHA Class III/IV (1-year, 13.4% vs 17.3% with no iASD closure; *P* = .36) or KCCQ-OS score (Table 3).

Significant underlying pulmonary hypertension is often cited as an indication for closing an iASD during a TS procedure. However, findings from the pulmonary hypertension subgroup analysis revealed higher 30-day mortality and increased cardiac readmissions at 1 year in the iASD closure group compared to those without closure. These results suggest that closing an iASD solely to address pulmonary hypertension may not confer clinical benefit and could potentially lead to adverse outcomes in this patient population.

Ideally, a randomized controlled trial comparing iASD closure vs no closure would be performed, but this is not ethically feasible in the general MViV population, given general views indicating the need to perform iASD closure usually when shunting is apparent. However, a randomized analysis might be feasible for patients without a clear indication of iASD closure. Accordingly, we consider our analysis to be the best available approach for the evaluation of iASD closure outcomes for patients who underwent TS MViV. Our more focused analysis, restricting without closure to sites that simply do not perform any closures as a means of limiting patient selection bias, supports that the iASD procedure is a safe procedure without overt complications or worsened outcomes.

Transseptal mitral valve implantation with large-bore sheaths requires large-diameter TS sheaths that commonly result in iASD, the majority of which resolve over time, but some may require closure.^8^ Closure of iASD following a TS MViV procedure is variable between US sites and is a relatively infrequent procedure. A series of retrospective studies have demonstrated potential negative impacts for persistent iASD.^9^ Toyama et al^10^ reported on a series of TEER patients and demonstrated that 23 of the 96 patients had a persistent iASD at 1 year. Significantly, TEER patients with a persistent iASD had worse clinical and echocardiographic outcomes than those who did not. Heart failure readmissions (26% vs 2.7%; *P* < .05), the severity of TR, and right heart enlargement were significantly increased in this cohort of patients. In a smaller series of TEER patients from the EVEREST II trial, those with any iASD 1 year after TEER presented with larger left atrial volumes, less reduction in MR, and a higher grade of TR.^11^ The increase in iASD size for TS MViV may have additional clinical and echocardiographic implications compared to smaller TS procedures. Additionally, our analysis reveals a slight increase in the risk for stroke at 1-year end points for patients with a persistent iASD, but it was not statistically significant (Figure 3B).

To date, there are no guideline recommendations for the management of iASD.^12^ The most commonly reported reason for performing an iASD closure includes the risk of left-to-right shunting (TR progression and right heart failure) and right-to-left shunting (hypoxia and paradoxical emboli to address potential hypoxia).^13^ This clinical scenario is usually quickly identified and addressed before the patient is discharged from the hospital. Still, other clinical considerations for iASD closure are less well-defined. The MITHRAS trial randomized 80 patients to iASD closure or conservative therapy after TEER.^9^ At 6-month and 1-year follow-up, there was a significant reduction in hospitalization for the patients who underwent iASD closure vs those with conservative therapy.^9^^,^^14^ Compared to TEER, the septostomy created during a TS MViV procedure tends to be larger, with 12 to 14 mm balloons commonly used to create the defect.^15^

### Study limitations

Several inherent limitations in this study primarily stem from the fact this was an observational study dependent on national databases. Administrative databases may introduce errors in coding, data retrieving, and reporting. Additionally, there were no independent reviews of echocardiographic core laboratory imaging or independent adjudication of adverse events. Also, the STS/ACC TVT Registry database lacks a standard definition of left ventricular outflow tract obstruction. Importantly, the clinical reasoning and echocardiographic findings leading to the decision to perform closure of an iASD were not available for review.

Our study analyzed outcomes from 472 iASD closures from June 2015 to September 2023. Although the number of both TS MViV procedures and subsequent iASD closures has continuously increased since the 2017 FDA approval of SAPIEN 3 MViV procedures in high- or greater-risk patients, the closure procedure remains relatively infrequently used according to our analysis (8.8%). The pulmonary hypertension subgroup analysis was limited by the availability of right heart catheterization data, with only approximately 40% of patients (184 iASD closures) having submitted hemodynamic measurements.

The present study is the largest study on the closure of iASD to date, but larger numbers would help obtain more precise data with greater potential for revealing predictors of outcomes and events after performing multivariable analysis. Finally, we do not have follow-up echocardiograms in order to evaluate spontaneous iASD closure rates and changes in RA/RV dimensions.

## Conclusion

Our analysis indicates that iASD closure is a safe and well-tolerated procedure with potential benefits for improving echocardiographic and clinical outcomes. The study supports that transcatheter MViV implantation using BEV is associated with high procedural success, low complication rates, and lower 30-day mortality rates than those predicted by the STS score independent of performing iASD closure. Most patients experienced significant improvement in symptoms and quality of life (NHYA and KCCQ-OS scores), which were maintained at 1 year. Concomitant iASD closure during a TS MViV procedure is infrequently performed in the United States, but iASD closure should be considered in carefully selected patients. Further research is warranted to better understand the risks and benefits of iASD closure in the context of pulmonary hypertension.
