# Supplementary material for: Clinical Outcomes for Closure of Iatrogenic Atrial Septal Defects Following Transseptal SAPIEN Mitral Valve-in-Valve Procedures
Source: J Soc Cardiovasc Angiogr Interv. 2025 Jun 17;4(6):102636. doi: 10.1016/j.jscai.2025.102636 (PMC12230488; doi:10.1016/j.jscai.2025.102636)
Supplement: Supplementary Tables 1-7 [file mmc1.docx]

**SUPPLEMENTARY MATERIAL: Sensitivity Analysis: MViV + ASD Closure vs MViV + No ASD Closure (in sites that *did* perform ASD closures).**

**Supplemental Table S1. Unmatched and matched baseline clinical and echocardiographic characteristics.**

|  | **All MViV Patients**  **(N=3348)** | **Unmatched**  **MViV + ASD Closure**  **(n=472)** | **Unmatched**  **MViV + No ASD Closure**  **(n=2876)** | **P-value** | **Matched**  **MViV + ASD Closure**  **(n=472)** | **Matched**  **MViV + No ASD Closure**  **(n=472)** | **P-value** |
| --- | --- | --- | --- | --- | --- | --- | --- |
| **Clinical Characteristics** |  |  |  |  |  |  |  |
| Age (years) | 72.6 ± 11.8 (3348) | 72.9 ± 11.6 (472) | 72.6 ± 11.9 (2876) | 0.62 | 72.9 ± 11.6 (472) | 72.9 ± 11.7 (472) | 0.98 |
| STS risk score (%) | 9.2 ± 8.3 (3126) | 10.5 ± 9.0 (438) | 9.0 ± 8.2 (2688) | 0.0008 | 10.5 ± 9.0 (438) | 10.5 ± 8.9 (446) | 1 |
| Male | 41.8 (1399/3348) | 34.5 (163/472) | 43.0 (1236/2876) | 0.0006 | 34.5 (163/472) | 35.0 (165/472) | 0.89 |
| Female | 58.2  (1949/3348) | 65.5  (309/472) | 57.0  (1640/2876) | 0.0006 | 65.5 (309/472) | 65.0 (307/472) | 0.89 |
| BMI (kg/m^2^) | 27.6 ± 15.7 (3340) | 27.3 ± 6.7 (472) | 27.6 ± 16.7 (2868) | 0.5 | 27.3 ± 6.7 (472) | 27.0 ± 6.3 (470) | 0.5 |
| Hypertension | 83.6 (2798/3348) | 87.5  (413/472) | 82.9 (2385/2876) | 0.01 | 87.5 (413/472) | 86.9 (410/472) | 0.77 |
| Diabetes | 27.2 (911/3346) | 28.8 (136/472) | 27.0 (775/2874) | 0.4 | 28.8 (136/472) | 29.0 (137/472) | 0.94 |
| Currently on dialysis | 4.7  (156/3345) | 6.8 (32/472) | 4.3 (124/2873) | 0.02 | 6.8 (32/472) | 5.9 (28/472) | 0.59 |
| Peripheral arterial disease | 15.1 504/3345 | 15.1 (71/471) | 15.1 (433/2874) | 1 | 15.1 (71/471) | 15.7 (74/472) | 0.8 |
| Home oxygen | 12.8 (428/3346) | 15.7 (74/471) | 12.3 354/2875 | 0.04 | 15.7 (74/471) | 15.0 (71/472) | 0.78 |
| Cardiogenic Shock within 24 hours | 4.5 (151/3346) | 8.9 (42/472) | 3.8 (109/2874) | <0.0001 | 8.9 (42/472) | 8.9 (42/472) | 1 |
| Carotid stenosis | 10.0 (270/2690) | 9.6 (38/396) | 10.1 (232/2294) | 0.75 | 9.6 (38/396) | 8.0 (29/362) | 0.44 |
| Atrial fibrillation/flutter | 73.7 (2468/3347) | 78.4 (370/472) | 73.0 (2098/2875) | 0.01 | 78.4 (370/472) | 80.0 (377/472) | 0.58 |
| Prior stroke | 17.8 (595/3347) | 16.7 (79/472) | 18.0 (516/2875) | 0.52 | 16.7 (79/472) | 16.1 (76/472) | 0.79 |
| Chronic lung disease | 38.1 (1268/3329) | 43.1 (203/471) | 37.3 (1065/2858) | 0.02 | 43.1 (203/471) | 40.6 (189/466) | 0.43 |
| Prior PCI | 15.7 (525/3343) | 18.4 (87/472) | 15.3 (438/2871) | 0.08 | 18.4 (87/472) | 18.3 (86/471) | 0.95 |
| Prior CABG | 29.7 (992/3342) | 30.2 (142/471) | 29.6 (850/2871) | 0.81 | 30.2 (142/471) | 31.6 (149/471) | 0.62 |
| Porcelain aorta | 0.5 (15/3344) | 0.0 (0/472) | 0.5 (15/2872) | 0.15 | 0.0 (0/472) | 0.0 (0/472) | N/A |
| BNP | 791.6 ± 1034.5 (1477) | 1019.8 ± 1171.6 (208) | 754.2 ± 1005.9 (1269) | 0.002 | 1019.8 ± 1171.6 (208) | 889.0 ± 1040.0 (198) | 0.23 |
| GFR (mL/min/1.73 m^2^) | 56.6 ± 25.8 (3334) | 53.0 ± 29.3 (471) | 57.2 ± 25.1 (2863) | 0.004 | 53.0 ± 29.3 (471) | 53.8 ± 23.9 (469) | 0.65 |
| Hemoglobin | 11.6 ± 2.1 (3333) | 11.2 ± 2.0 (471) | 11.6 ± 2.1 (2862) | 0.0002 | 11.2 ± 2.0 (471) | 11.3 ± 2.1 (470) | 0.65 |
| NYHA III/IV | 81.6 (2682/3288 | 85.7 (402/469) | 80.9 (2280/2819) | 0.01 | 85.7 (402/469) | 85.4 (397/465) | 0.88 |
| KCCQ-OS | 37.2 ± 24.1 (2789) | 32.2 ± 23.0 (380) | 38.0 ± 24.1 (2409) | <0.0001 | 32.2 ± 23.0 (380) | 33.2 ± 23.6 (386) | 0.54 |
| **Echocardiography Characteristics** |  |  |  |  |  |  |  |
| Left ventricle ejection fraction, % | 55.3 ± 11.7 (3316) | 54.6 ± 12.2 (470) | 55.4 ± 11.6 (2846) | 0.15 | 54.6 ± 12.2 (470) | 54.5 ± 12.4 (466) | 0.88 |
| Mitral mean valve gradient, mmHg | 13.2 ± 6.4 (3204) | 13.6 ± 5.8 (457) | 13.1 ± 6.4 (2747) | 0.15 | 13.6 ± 5.8 (457) | 13.5 ± 6.3 (455) | 0.79 |
| Mitral valve area, cm^2^ | 1.4 ± 1.0 (2407) | 1.3 ± 0.9 (358) | 1.4 ± 1.0 (2049) | 0.17 | 1.3 ± 0.9 (358) | 1.5 ± 1.3 (333) | 0.05 |
| ≥ Moderate aortic regurgitation | 9.5 (315/3326) | 8.7 (41/469) | 9.6 (274/2857) | 0.56 | 8.7 (41/469) | 9.0 (42/468) | 0.9 |
| ≥ Moderate mitral regurgitation | 55.0 (1824/3319) | 57.0 (268/470) | 54.6 (1556/2849) | 0.33 | 57.0 (268/470) | 55.0 (255/464) | 0.53 |
| ≥ Moderate tricuspid regurgitation | 57.3 (1909/3332) | 68.3 (319/467) | 55.5 (1590/2865) | <0.0001 | 68.3 (319/467) | 70.8 (332/469) | 0.41 |
| Pulmonary capillary wedge pressure (mmHg) | 26.2 ± 8.5 (1608) | 26.7 ± 9.3 (259) | 26.1 ± 8.3 (1349) | 0.32 | 26.7 ± 9.3 (259) | 26.9 ± 8.7 (218) | 0.84 |
| Pulmonary artery pressure (mean) mmHg | 40.5 ± 12.1 (1686) | 42.5 ± 11.6 (261) | 40.1 ± 12.2 (1425) | 0.003 | 42.5 ± 11.6 (261) | 43.2 ± 12.5 (234) | 0.54 |
| Pulmonary artery pressure (systolic) mmHg | 62.2 ± 18.7 (1852) | 65.8 ± 18.2 (288) | 61.5 ± 18.7 (1564) | 0.0003 | 65.8 ± 18.2 (288) | 66.1 ± 19.0 (266) | 0.89 |
| Right atrial pressure/Central venous pressure, mmHg | 12.0 ± 6.6 (1838) | 14.5 ± 7.3 (280) | 11.6 ± 6.4 (1558) | <0.0001 | 14.5 ± 7.3 (280) | 13.5 ± 6.6 (256) | 0.13 |
| Pulmonary vascular resistance | 304.9 ± 286.1 (1425) | 354.5 ± 297.3 (229) | 295.4 ± 283.0 (1196) | 0.004 | 354.5 ± 297.3 (229) | 366.9 ± 317.3 (194) | 0.68 |

Values are mean ± SD (n) or % (n/N)

Abbreviations: BMI, body mass index; BNP, B-type natriuretic peptide; CABG, coronary artery bypass grafting; GFR, glomerular filtration rate; KCCQ-OS, Kansas City Cardiomyopathy Questionnaire - Overall Summary Score; NYHA, New York Heart Association; PCI, percutaneous coronary intervention; STS, Society of Thoracic Surgeons.

**Supplemental Table S2. Propensity-matched procedural and in-hospital outcomes.**

|  | **MViV + ASD Closure**  **(n=472)** | **MViV + No ASD Closure**  **(n=472)** | **P-value** |
| --- | --- | --- | --- |
| **Procedural Outcomes** |  |  |  |
| MVARC technical success^a^ | 95.8 (452/472) | 96.6 (456/472) | 0.61 |
| Procedure status |  |  |  |
| Elective | 62.3 (294/472) | 62.0 (292/471) | 0.93 |
| Urgent | 33.7 (159/472) | 34.4 (162/471) | 0.82 |
| Emergency | 3.0 (14/472) | 3.2 (15/471) | 0.85 |
| Salvage | 1.1 (5/472) | 0.4 (2/471) | 0.45 |
| THV type |  |  |  |
| SAPIEN 3 Ultra Resilia | 7.2 (34/472) | 6.4 (30/472) | 0.60 |
| SAPIEN 3 Ultra | 26.9 (127/472) | 23.3 (110/472) | 0.20 |
| SAPIEN 3 | 65.9 (311/472) | 70.3 (332/472) | 0.14 |
| THV size |  |  |  |
| 20 mm | 0.0 (0/472) | 0.2 (1/472) | 1.00 |
| 23 mm | 6.8 (32/472) | 8.7 (41/472) | 0.27 |
| 26 mm | 38.4 (181/472) | 48.1 (227/472) | 0.003 |
| 29 mm | 54.9 (259/472) | 43.0 (203/472) | 0.0003 |
| Device implant success | 96.4 (455/472) | 97.5 (460/472) | 0.35 |
| Procedure time, min | 116.8 ± 64.9 (471) | 104.4 ± 61.6 (471) | 0.003 |
| Fluoroscopy time, min | 37.5 ± 23.8 (420) | 32.6 ± 24.4 (429) | 0.004 |
| Procedure aborted | 0.2 (1/472) | 0.6 (3/472) | 0.62 |
| Converted to open heart surgery | 0.6 (3/472) | 0.6 (3/472) | 1 |
| Cardiopulmonary bypass | 1.4 (6/442) | 1.3 (6/449) | 0.98 |
| Mechanical support | 5.5 (26/472) | 5.9 (28/472) | 0.78 |
| Device embolization | 0.4 (2/472) | 0.0 (0/472) | 0.5 |
| Device thrombosis | 0.2 (1/472) | 0.2 (1/472) | 1 |
| **In-Hospital Outcomes** |  |  |  |
| All-cause death | 5.5 (26/472) | 4.7 (22/472) | 0.55 |
| Cardiovascular death | 3.0 (14/472) | 2.8 (13/472) | 0.85 |
| Stroke | 1.3 (6/472) | 0.9 (4/472) | 0.75 |
| Mitral valve reintervention | 0.6 (3/472) | 0.0 (0/472) | 0.25 |
| LVOT obstruction | 0.9 (4/472) | 0.4 (2/472) | 0.69 |
| New pacemaker without baseline pacemaker | 1.5 (5/324) | 1.2 (4/335) | 0.75 |
| Periprocedural myocardial infarction | 0.2 (1/472) | 0.2 (1/472) | 1.00 |
| Device thrombosis | 0.2 (1/472) | 0.4 (2/472) | 1.00 |
| Major vascular complication | 1.5 (7/472) | 0.9 (4/472) | 0.36 |
| Cardiac perforation | 0.2 (1/472) | 1.3 (6/472) | 0.12 |
| Length of stay, median (IQR) | 3.0 [1.0, 7.0] | 2.0 [1.0, 6.0] | 0.07 |
| Discharged home | 78.0 (368/472) | 80.5 (380/472) | 0.34 |
| Discharge medication |  |  |  |
| Antiplatelet | 66.9 (293/438) | 70.8 (312/441) | 0.22 |
| Anticoagulant | 85.4 (374/438) | 82.3 (363/441) | 0.22 |

Values are mean ± SD (n) or % (n/N)

Abbreviations: IQR, interquartile range; LVOT, left ventricular outflow tract; MVARC, Mitral Valve Academic Research Consortium; NA, not applicable; THV, transcatheter heart valve

^a^MVARC technical success was defined as at exit from the hybrid suite, patient is alive with successful access, delivery, and retrieval of the device delivery system, successful deployment and correct position of the first intended device, and freedom from emergency surgery or reintervention associated with the device or access procedure.

**Supplemental Table S3. Propensity-matched 30-day and 1-year outcomes.**

|  | **30-Day**  **MViV + ASD Closure**  **(n=472)** | **30-Day**  **MViV + No ASD Closure**  **(n=472)** | **P-value** | **1-Year**  **MViV + ASD Closure**  **(n=472)** | **1-Year**  **MViV + No ASD Closure**  **(n=472)** | **P-value** |
| --- | --- | --- | --- | --- | --- | --- |
| **Clinical Outcomes** |  |  |  |  |  |  |
| All-cause death | 7.2 (33) | 6.3 (29) | 0.60 | 19.3 (72) | 16.1 (62) | 0.25 |
| Cardiac death | 3.3 (15) | 3.6 (16) | 0.86 | 6.1 (23) | 5.0 (20) | 0.61 |
| Observed:expected ratio | 0.72 | 0.62 |  | NA | | |
| Stroke | 1.7 (8) | 1.8 (8) | 0.99 | 3.3 (12) | 3.5 (13) | 0.93 |
| Mitral valve reintervention | 0.9 (4) | 0.0 (0) | 0.04 | 1.3 (5) | 0.8 (2) | 0.23 |
| Readmission for heart failure | 3.9 (17) | 3.0 (13) | 0.44 | 13.0 (43) | 13.1 (41) | 0.69 |
| New requirement for dialysis | 2.4 (11) | 1.8 (8) | 0.49 | 2.7 (12) | 2.2 (9) | 0.50 |
| Major vascular complication | 1.5 (7) | 0.9 (4) | 0.36 | 1.8 (8) | 1.9 (7) | 0.78 |
| Myocardial infarction | 0.5 (2) | 0.4 (2) | 1.00 | 0.9 (3) | 0.8 (3) | 0.98 |
| New onset of atrial fibrillation | 1.5 (7) | 2.0 (9) | 0.61 | 2.2 (10) | 2.5 (11) | 0.80 |
| New pacemaker without baseline pacemaker | 1.6 (5) | 1.3 (4) | 0.70 | 2.8 (7) | 1.8 (5) | 0.49 |
| Device thrombosis | 0.5 (2) | 0.4 (2) | 1.00 | 0.8 (3) | 0.7 (3) | 0.99 |
| NYHA III/IV | 12.7 (33/259) | 13.8 (41/298) | 0.72 | 14.1 (19/135) | 16.4 (24/146) | 0.58 |
| KCCQ improvement from baseline | 35.0 ± 28.7 (249) | 37.7 ± 29.1 (249) | 0.30 | 42.5 ± 28.5 (115) | 43.7 ± 29.9 (132) | 0.74 |
| **Echocardiographic Outcomes** |  |  |  |  |  |  |
| Left ventricular ejection fraction, % | 53.9 ± 11.5 (308) | 53.1 ± 12.3 (309) | 0.39 | 53.3 ± 12.3 (116) | 52.2 ± 12.6 (146) | 0.48 |
| Mean mitral valve gradient, mmHg | 7.7 ± 3.0 (303) | 7.4 ± 2.6 (302) | 0.21 | 7.6 ± 3.3 (109) | 7.5 ± 3.7 (145) | 0.78 |
| Mitral valve area, cm^2^ | 1.9 ± 1.2 (175) | 1.9 ± 1.1 (185) | 0.75 | 1.9 ± 1.2 (57) | 1.9 ± 0.9 (86) | 0.91 |
| ≥ Moderate mitral regurgitation | 1.0 (3/307) | 0.3 (1/308) | 0.37 | 0.0 (0/114) | 1.3 (2/149) | 0.51 |
| ≥ Moderate tricuspid regurgitation | 46.1 (142/308) | 43.1 (132/306) | 0.46 | 36.8 (42/114) | 46.3 (68/147) | 0.13 |

Values are Kaplan-Meier estimate % (No. of events), mean ± SD (n) or % (n/N)

Abbreviations: KCCQ, Kansas City Cardiomyopathy Questionnaire; KM, Kaplan-Meier; NYHA, New York Heart Association; SD, standard deviation.

**SUPPLEMENTARY MATERIAL:** **Subgroup analysis: MViV + ASD Closure vs MViV + No ASD Closure in patients with mean pulmonary artery pressure ≥35 mmHg (in sites that *did not* perform ASD closures).**

**Supplemental Table S4.** **Propensity-matched baseline clinical and echocardiographic characteristics in patients with mean pulmonary artery pressure ≥35 mmHg.**

|  | **ASD Closure within 30 Days (n=184)** | **No ASD Closure in No ASD Site (n=184)** | **P-value** |
| --- | --- | --- | --- |
| **Clinical characteristic** |  |  |  |
| Age (years) | 71.6 ± 11.4 (184) | 71.1 ± 11.4 (184) | 0.67 |
| Female | 65.8% (121/184) | 65.2% (120/184) | 0.91 |
| STS score (%) | 10.9 ± 8.8 (171) | 10.1 ± 7.4 (168) | 0.38 |
| BMI (kg/m^2^) | 28.0 ± 6.8 (184) | 27.9 ± 6.2 (184) | 0.91 |
| Permanent pacemaker | 33.2% (61/184) | 34.8% (64/184) | 0.74 |
| Previous ICD | 13.0% (24/184) | 14.1% (26/184) | 0.76 |
| Prior MI | 23.4% (43/184) | 17.4% (32/184) | 0.15 |
| Prior PCI | 16.8% (31/184) | 15.2% (28/184) | 0.67 |
| Prior CABG | 32.2% (59/183) | 29.9% (55/184) | 0.63 |
| Prior aortic valve procedure | 26.1% (48/184) | 21.9% (40/183) | 0.34 |
| Prior stroke | 15.2% (28/184) | 13.6% (25/184) | 0.66 |
| Prior TIA | 9.8% (18/184) | 6.5% (12/184) | 0.25 |
| Hypertension | 87.0% (160/184) | 87.0% (160/184) | 1.00 |
| Immunocompromised | 9.8% (18/184) | 9.5% (17/179) | 0.93 |
| Endocarditis | 12.5% (23/184) | 12.0% (22/184) | 0.87 |
| Diabetes mellitus | 29.9% (55/184) | 37.0% (68/184) | 0.15 |
| Peripheral arterial disease | 16.3% (30/184) | 14.1% (26/184) | 0.56 |
| GFR | 49.9 ± 23.3 (184) | 48.6 ± 20.7 (184) | 0.58 |
| Currently on dialysis | 6.5% (12/184) | 6.6% (12/183) | 0.99 |
| Hostile chest | 12.5% (23/184) | 14.1% (26/184) | 0.65 |
| Carotid stenosis | 12.5% (19/152) | 11.3% (18/160) | 0.73 |
| Heart failure hospitalization within past year | 68.5% (111/162) | 72.4% (110/152) | 0.46 |
| Cardiogenic shock within 24 hours | 10.9% (20/184) | 9.8% (18/184) | 0.73 |
| Porcelain aorta | 0.0% (0/184) | 0.0% (0/184) | N/A |
| Atrial fibrillation/flutter | 81.5% (150/184) | 81.0% (149/184) | 0.89 |
| Chronic lung disease | 49.7% (91/183) | 53.6% (98/183) | 0.46 |
| Home oxygen | 20.7% (38/184) | 24.0% (44/183) | 0.44 |
| Positive inotropes | 15.2% (28/184) | 19.1% (35/183) | 0.32 |
| BNP | 1056.7 ± 1011.0 (85) | 1091.1 ± 1035.3 (63) | 0.84 |
| NT proBNP | 6708.9 ± 9410.0 (74) | 6767.5 ± 8529.2 (85) | 0.97 |
| Hemoglobin | 11.0 ± 1.9 (184) | 10.8 ± 1.9 (183) | 0.56 |
| **Operator reason for procedure** |  |  | 0.16 |
| Inoperable/Extreme risk | 23.4% (43/184) | 16.4% (30/183) | 0.09 |
| High risk | 65.2% (120/184) | 66.7% (122/183) | 0.77 |
| Intermediate risk | 9.2% (17/184) | 15.3% (28/183) | 0.08 |
| Low risk | 2.2% (4/184) | 1.6% (3/183) | 1.00 |
| NYHA class III/IV | 90.8% (167/184) | 90.1% (164/182) | 0.83 |
| KCCQ-OS | 28.0 ± 20.7 (153) | 26.8 ± 20.8 (148) | 0.62 |
| **Echocardiographic characteristic** |  |  |  |
| Left main stenosis ≥ 50% | 4.6% (8/174) | 5.1% (9/175) | 0.81 |
| Mitral annular calcification | 50.8% (33/65) | 48.1% (26/54) | 0.78 |
| MV area (cm^2^) | 1.2 ± 0.9 (150) | 1.4 ± 1.0 (143) | 0.24 |
| MV mean gradient (mmHg) | 14.5 ± 6.2 (180) | 14.4 ± 6.6 (179) | 0.95 |
| LVEF (%) | 54.2 ± 13.0 (183) | 53.5 ± 13.4 (183) | 0.62 |
| ≥Moderate mitral regurgitation | 50.5% (93/184) | 47.8% (87/182) | 0.60 |
| Mitral Stenosis | 86.7% (157/181) | 82.5% (146/177) | 0.26 |
| ≥Moderate aortic regurgitation | 10.4% (19/183) | 10.9% (20/184) | 0.88 |
| Aortic stenosis | 20.9% (38/182) | 17.1% (31/181) | 0.36 |
| ≥Moderate tricuspid regurgitation | 70.1% (129/184) | 69.6% (128/184) | 0.91 |
| Pulmonary capillary wedge pressure (mmHg) | 29.3 ± 8.8 (173) | 29.6 ± 8.6 (170) | 0.81 |
| Mean pulmonary artery pressure (mmHg) | 47.4 ± 8.6 (184) | 47.4 ± 10.1 (184) | 0.97 |
| Right atrial pressure/CVP (mmHg) | 16.0 ± 6.6 (175) | 16.2 ± 7.0 (174) | 0.87 |
| Pulmonary vascular resistance | 414.7 ± 279.6 (161) | 411.6 ± 454.2 (149) | 0.94 |

Values are mean ± SD (n) or % (n/N)

Abbreviations: STS, Society of Thoracic Surgeons; BMI, body mass index; ICD, implantable cardioverter defibrillator; MI, myocardial infarction; PCI, percutaneous coronary intervention; CABG, coronary artery bypass grafting; TIA, transient ischemic attack; GFR, glomerular filtration rate; BNP, B-type natriuretic peptide; NT proBNP, N-terminal pro-B-type natriuretic peptide; NYHA, New York Heart Association; KCCQ-OS, Kansas City Cardiomyopathy Questionnaire - Overall Summary Score; MV, mitral valve; LVEF, left ventricular ejection fraction; CVP, central venous pressure.

**Supplemental Table S5. Propensity-matched procedural and in-hospital outcomes in patients with mean pulmonary artery pressure ≥35 mmHg.**

|  | **ASD Closure within 30 Days (n=184)** | **No ASD Closure in No ASD Site (n=184)** | **P-value** |
| --- | --- | --- | --- |
| **Procedural Outcomes** |  |  |  |
| Device implanted successfully | 96.2% (177/184) | 97.3% (179/184) | 0.56 |
| Procedure status |  |  | 0.61 |
| Elective | 59.8% (110/184) | 63.6% (117/184) | 0.45 |
| Urgent | 35.9% (66/184) | 31.0% (57/184) | 0.32 |
| Emergency | 2.2% (4/184) | 3.8% (7/184) | 0.36 |
| Salvage | 2.2% (4/184) | 1.6% (3/184) | 1.00 |
| *Anesthesia type* |  |  | 0.02 |
| General anesthesia | 90.2% (166/184) | 97.3% (179/184) | 0.005 |
| Moderate sedation | 9.2% (17/184) | 2.7% (5/184) | 0.008 |
| Conversion to open heart surgery | 1.6% (3/184) | 1.6% (3/184) | 1.00 |
| Total procedure time (min) | 127.5 ± 79.3 (183) | 113.5 ± 62.4 (184) | 0.06 |
| Fluoroscopy time (min) | 38.0 ± 24.3 (176) | 33.0 ± 16.6 (174) | 0.02 |
| Contrast volume (ml) | 17.6 ± 33.2 (170) | 15.6 ± 29.9 (155) | 0.57 |
| *THV type* |  |  |  |
| S3 | 65.8% (121/184) | 65.8% (121/184) | 1.00 |
| S3 Ultra | 29.9% (55/184) | 30.4% (56/184) | 0.91 |
| S3 Ultra Resilia | 4.3% (8/184) | 3.8% (7/184) | 0.79 |
| *THV size* |  |  | 0.37 |
| 20 mm | 0.0% (0/184) | 0.0% (0/184) | N/A |
| 23 mm | 6.0% (11/184) | 9.8% (18/184) | 0.18 |
| 26 mm | 44.6% (82/184) | 44.6% (82/184) | 1.00 |
| 29 mm | 49.5% (91/184) | 45.7% (84/184) | 0.46 |
| Bioprosthetic valve fracture attempted | 7.5% (7/93) | 5.8% (6/104) | 0.62 |
| Mechanical support | 7.6% (14/184) | 6.5% (12/184) | 0.68 |
| Procedure aborted | 0.5% (1/184) | 0.5% (1/184) | 1.00 |
| **Procedure complication** |  |  |  |
| Transseptal complication | 4.3% (8/184) | 0.5% (1/184) | 0.04 |
| Device embolization | 0.5% (1/184) | 0.0% (0/184) | 1.00 |
| Device migration | 0.0% (0/184) | 0.5% (1/184) | 1.00 |
| Procedure mortality | 0.0% (0/184) | 1.1% (2/184) | 0.50 |
| Length of stay (days) | 3.0 [1.5, 7.5] | 3.0 [1.0, 6.0] | 0.07 |
| ICU length of stay (hours) | 24.0 [2.9, 54.5] | 25.9 [4.0, 48.0] | 0.86 |
| Discharged w/ anticoagulants | 88.4% (145/164) | 85.5% (147/172) | 0.42 |
| Discharged w/ antiplatelets | 68.9% (113/164) | 75.6% (130/172) | 0.17 |
| Discharged home | 78.3% (144/184) | 83.2% (153/184) | 0.23 |

Values are mean ± SD (n) or % (n/N). Abbreviations: THV, transcatheter heart valve; S3, SAPIEN 3.

**Supplemental Table S6. Propensity-matched echocardiographic outcomes at discharge, 30 days and one year in patients with mean pulmonary artery pressure ≥35 mmHg.**

|  | **ASD Closure within 30 Days (n=184)** | **No ASD Closure in No ASD Site (n=184)** | **P-value** |
| --- | --- | --- | --- |
| **Discharge** |  |  |  |
| MV area (cm^2^) | 2.0 ± 1.2 (90) | 2.2 ± 0.9 (102) | 0.44 |
| MV mean gradient (mmHg) | 6.7 ± 2.9 (166) | 6.8 ± 2.7 (169) | 0.69 |
| ≥Moderate PVL | 0.0% (0/138) | 0.0% (0/132) | N/A |
| ≥Moderate mitral regurgitation | 0.0% (0/167) | 0.0% (0/171) | N/A |
| ≥Moderate tricuspid regurgitation | 52.4% (44/84) | 50.5% (48/95) | 0.80 |
| **30 days** |  |  |  |
| MV area (cm^2^) | 2.0 ± 1.4 (69) | 2.0 ± 0.9 (64) | 0.81 |
| MV mean gradient (mmHg) | 8.1 ± 3.1 (113) | 7.9 ± 2.7 (110) | 0.59 |
| LVEF (%) | 55.1 ± 11.4 (115) | 54.1 ± 11.8 (112) | 0.51 |
| ≥Moderate PVL | 0.0% (0/88) | 0.0% (0/89) | N/A |
| ≥Moderate mitral regurgitation | 2.7% (3/113) | 0.0% (0/112) | 0.25 |
| ≥Moderate tricuspid regurgitation | 43.1% (50/116) | 53.6% (60/112) | 0.11 |
| **1 year** |  |  |  |
| MV area (cm^2^) | 1.6 ± 0.6 (26) | 1.9 ± 1.3 (34) | 0.24 |
| MV mean gradient (mmHg) | 8.6 ± 4.1 (46) | 7.4 ± 3.1 (54) | 0.12 |
| LVEF (%) | 54.7 ± 12.9 (48) | 56.0 ± 10.6 (60) | 0.59 |
| ≥Moderate PVL | 0.0% (0/40) | 0.0% (0/46) | N/A |
| ≥Moderate mitral regurgitation | 0.0% (0/46) | 0.0% (0/57) | N/A |
| ≥Moderate tricuspid regurgitation | 33.3% (16/48) | 47.5% (28/59) | 0.14 |

Values are mean ± SD (n) or % (n/N)

Abbreviations: MV, mitral valve; PVL, paravalvular leak; LVEF, left ventricular ejection fraction.

**Supplemental Table S7. Propensity-matched in-hospital, 30-day and one-year clinical outcomes in patients with mean pulmonary artery pressure ≥35 mmHg.**

|  | **ASD Closure within 30 Days (n=184)** | **No ASD Closure in No ASD Site (n=184)** | **P-value** |
| --- | --- | --- | --- |
| **In hospital** |  |  |  |
| All-cause mortality | 8.2% (15/184) | 4.9% (9/184) | 0.21 |
| Cardiac death | 3.3% (6/184) | 2.2% (4/184) | 0.75 |
| Mitral valve re-intervention | 0.0% (0/184) | 0.5% (1/184) | 1.00 |
| Major vascular complication | 1.6% (3/184) | 0.5% (1/184) | 0.62 |
| Life-threatening bleeding | 3.8% (7/184) | 1.1% (2/184) | 0.17 |
| New requirement for dialysis | 4.3% (8/184) | 1.6% (3/184) | 0.13 |
| Myocardial Infarction | 0.5% (1/184) | 0.0% (0/184) | 1.00 |
| New pacemaker w/o baseline pacemaker | 0.8% (1/123) | 2.5% (3/120) | 0.37 |
| New onset of atrial fibrillation | 1.6% (3/184) | 0.5% (1/184) | 0.62 |
| Stroke | 0.5% (1/184) | 1.6% (3/184) | 0.62 |
| TIA | 0.0% (0/184) | 0.5% (1/184) | 1.00 |
| LVOT obstruction | 1.1% (2/184) | 0.0% (0/184) | 0.50 |
| Device thrombosis | 0.0% (0/184) | 0.0% (0/184) | N/A |
| Cardiac perforation | 0.0% (0/184) | 1.1% (2/184) | 0.50 |
| ASD closure | 99.5% (183/184) | 0.0% (0/184) | <0.0001 |
| Any readmission | 0.0% (0/184) | 0.5% (1/184) | 1.00 |
| Cardiac readmission | 0.0% (0/184) | 0.0% (0/184) | N/A |
| Heart failure readmission | 0.0% (0/184) | 0.0% (0/184) | N/A |
| **30 days** |  |  |  |
| All-cause mortality | 9.7% (17) | 3.9% (7) | 0.03 |
| Cardiac death | 3.5% (6) | 1.6% (3) | 0.30 |
| Mitral valve re-intervention | 0.7% (1) | 0.5% (1) | 0.98 |
| Major vascular complication | 1.6% (3) | 0.5% (1) | 0.32 |
| Life-threatening bleeding | 3.0% (5) | 0.6% (1) | 0.09 |
| New requirement for dialysis | 4.6% (8) | 1.7% (3) | 0.12 |
| Myocardial Infarction | 0.6% (1) | 0.0% (0) | 0.30 |
| New pacemaker w/o baseline pacemaker | 0.8% (1) | 2.5% (3) | 0.30 |
| New onset of atrial fibrillation | 1.7% (3) | 0.6% (1) | 0.31 |
| Stroke | 0.5% (1) | 1.7% (3) | 0.33 |
| TIA | 0.6% (1) | 0.5% (1) | 0.98 |
| LVOT obstruction | 1.1% (2) | 0.0% (0) | 0.16 |
| Device thrombosis | 0.0% (0) | 0.0% (0) | N/A |
| ASD closure | 100.0% (184) | 0.0% (0) | <0.0001 |
| Any readmission | 12.8% (21) | 9.3% (16) | 0.28 |
| Cardiac readmission | 1.2% (2) | 1.2% (2) | 0.94 |
| Heart failure readmission | 4.8% (8) | 2.9% (5) | 0.34 |
| **1 year** |  |  |  |
| All-cause mortality | 20.4% (31) | 16.4% (22) | 0.19 |
| Cardiac death | 6.9% (10) | 5.1% (7) | 0.44 |
| Mitral valve re-intervention | 0.7% (1) | 2.5% (3) | 0.32 |
| Major vascular complication | 1.6% (3) | 0.5% (1) | 0.32 |
| Life-threatening bleeding | 6.6% (9) | 4.2% (5) | 0.25 |
| New requirement for dialysis | 5.3% (9) | 2.6% (4) | 0.15 |
| Myocardial Infarction | 0.6% (1) | 0.0% (0) | 0.30 |
| New pacemaker w/o baseline pacemaker | 3.7% (3) | 4.3% (4) | 0.66 |
| New onset of atrial fibrillation | 1.7% (3) | 1.9% (3) | 0.95 |
| Stroke | 0.5% (1) | 3.7% (5) | 0.11 |
| TIA | 0.6% (1) | 0.5% (1) | 0.98 |
| LVOT obstruction | 1.1% (2) | 0.0% (0) | 0.16 |
| Device thrombosis | 0.0% (0) | 0.0% (0) | N/A |
| ASD closure | 100.0% (184) | 0.0% (0) | <0.0001 |
| Any readmission | 38.5% (50) | 34.5% (44) | 0.32 |
| Cardiac readmission | 14.1% (17) | 4.1% (5) | 0.008 |
| Heart failure readmission | 12.4% (17) | 15.0% (18) | 0.96 |

Values are % (n/N)

Abbreviations: TIA, transient ischemic attack; LVOT, left ventricular outflow tract; ASD, atrial septal defect.
